# Crowdsourcing in medical research: concepts and applications

**DOI:** 10.7717/peerj.6762

**Published:** 2019-04-12

**Authors:** Joseph D. Tucker, Suzanne Day, Weiming Tang, Barry Bayus

**Affiliations:** 1 Institute for Global Health and Infectious Diseases, University of North Carolina at Chapel Hill, Chapel Hill, NC, USA; 2 Faculty of Infectious and Tropical Diseases, London School of Hygiene & Tropical Medicine, University of London, London, UK; 3 Social Entrepreneurship to Spur Health (SESH) Global, Guangzhou, China; 4 Department of Social Medicine, University of North Carolina at Chapel Hill, Chapel Hill, NC, USA; 5 Department of STD Control, Dermatology Hospital of Southern Medical University, Guangzhou, China; 6 Kenan-Flagler School of Business, University of North Carolina at Chapel Hill, Chapel Hill, NC, USA

**Keywords:** Crowdsourcing, Theory, Literature review, Collective intelligence, Open source model

## Abstract

Crowdsourcing shifts medical research from a closed environment to an open collaboration between the public and researchers. We define crowdsourcing as an approach to problem solving which involves an organization having a large group attempt to solve a problem or part of a problem, then sharing solutions. Crowdsourcing allows large groups of individuals to participate in medical research through innovation challenges, hackathons, and related activities. The purpose of this literature review is to examine the definition, concepts, and applications of crowdsourcing in medicine. This multi-disciplinary review defines crowdsourcing for medicine, identifies conceptual antecedents (collective intelligence and open source models), and explores implications of the approach. Several critiques of crowdsourcing are also examined. Although several crowdsourcing definitions exist, there are two essential elements: (1) having a large group of individuals, including those with skills and those without skills, propose potential solutions; (2) sharing solutions through implementation or open access materials. The public can be a central force in contributing to formative, pre-clinical, and clinical research. A growing evidence base suggests that crowdsourcing in medicine can result in high-quality outcomes, broad community engagement, and more open science.

## Introduction

Crowdsourcing is an approach to problem solving that has gained momentum in the past decade (Han et al., 2018; Pan et al., 2017). Crowdsourcing involves an organization having a large group attempt to solve a problem or a component of a problem, then sharing solutions (Van Ess, 2010). This concept has facilitated ways for the public to engage in medical research, including innovation challenges (also called prize competitions, prize contests, or open contests), hackathons, online systems for collaboration, and other activities (Table 1) (Brabham et al., 2014; Pan et al., 2017; Ranard et al., 2014). We define medicine as the science and practice of preventing, diagnosing, and treating human disease (Oxford English Dictionary, 2019). Crowdsourcing is related to open innovation, diverging from conventional closed innovation medical research in several ways (Table 2) (Chesbrough, 2003).

**Table 1:** Crowdsourcing activities used to improve medical research: structure and function.

Crowdsourcing activity	Structure	Function
Innovation challenges	Open solicitation and promotion to the public for challenge submissions; evaluation, celebration, and sharing of challenge submissions	Generate innovative ideas, logos, images, or videos (e.g., images to increase HIV testing, strategies to promote hepatitis testing); accelerate pharmaceutical drug development
Hackathons	Short (often 3 days) event that brings together individuals around a common cause	Design a clinical algorithm, prevention service (e.g., design an HIV testing service), or new technology
Online collaboration systems	Websites or portals that allow individuals to solve a problem	Solve micro-tasks for a small amount of money (e.g., evaluation of surgical skills)

**Note:**

Crowdsourcing activities in medical research include innovation challenges, hackathons, and online collaboration systems.

**Table 2:** Comparison of conventional medical research and crowdsourced approaches.

	Conventional medical research (closed innovation)	Crowdsourced approach (open innovation)
Medical research questions	Those with medical research skills know best how to frame questions	A diverse group of individuals together know best how to frame questions
Methods for innovation	Internal teams led by experts, with little input from outside	Collaborative co-creation with non-experts and the public engaged
Intellectual property	Focus on controlling IP so that competitors will not benefit	Use others’ IP when it advances the research

**Note:**

Most medical research uses a framework of closed innovation (middle column). Crowdsourcing proposes an open innovation approach (right column).

Systematic reviews (Crequit et al., 2018; Ranard et al., 2014) and a World Health Organization practical guide on crowdsourcing (Han et al., 2018) demonstrate a growing evidence base supporting crowdsourcing in medicine. Some crowdsourcing projects have asked groups to develop health communication materials (e.g., images, videos) to promote HIV, hepatitis, and STI testing (Tang et al., 2018; Zhang et al., 2015, 2017b). Others have used crowdsourcing to accelerate antibiotic and other drug development (Desselle et al., 2017; Grammer et al., 2016; Shaw, 2017; Tufféry, 2015). However, this literature has not examined broader concepts and applications related to crowdsourcing in medicine.

The diversity of crowdsourcing approaches complicates attempts to achieve a single overarching conceptual framework (Ringh et al., 2015; Tang et al., 2016a). Some have suggested that crowdsourcing lacks a strong conceptual foundation (Geiger, Rosemann & Fielt, 2011). Others argue that the relatively brief history of crowdsourcing makes it premature to consider conceptual or theoretical elements (Geiger, Rosemann & Fielt, 2011). However, the conceptual basis of crowdsourcing reaches well beyond the first use of the term. This history alongside more recent data on collective intelligence and open-source models pave the way for a better understanding of crowdsourcing concepts and applications.

### Review methodology

This literature review examined the peer-reviewed and gray literature on crowdsourcing approaches related to medicine. We searched PubMed, Google Scholar, ResearchGate, and Academia.edu to identify potential studies for inclusion on February 25th, 2019. We focused on manuscripts that defined conceptual issues and applications of crowdsourcing for medical research. We excluded studies that were not in English. This manuscript defines crowdsourcing for medicine, identifies conceptual antecedents, considers relationships with other approaches, and examines common critiques.

### Crowdsourcing: a definition

There have been many definitions of crowdsourcing since Jeff Howe coined the term in 2005 (Brabham, 2008; Howe, 2006; Ranard et al., 2014; Tang et al., 2018; Wazny, 2017). The term is a portmanteau composed of “crowd” and “outsourcing.” The original definition was applied to describe companies outsourcing tasks to a group of individuals who worked collectively or individually. Howe himself realized that this initial definition was overly narrow and later expanded it to include the application of open-source principles to fields outside of software. However, this definition and many of the existing ones (Brabham, 2008; Ranard et al., 2014) do not include the subsequent obligation to share solutions. Van Ess (2010) suggested that crowdsourcing involves those with skills and those without skills attempting to solve a problem, then freely sharing some solutions with the public. We have included the sharing component for the following reasons: crowdsourcing activities draw on the strength of many laypeople who will not receive incentive prizes (e.g., gifts, money, mentorship, or other benefits); there are ethical problems with leveraging group insights (either individually or collectively) and not giving back to the group (Tucker et al., 2018); not sharing would likely diminish enthusiasm for sustained engagement from those who contribute to challenges; sharing may be more likely to advance medical knowledge. Many individuals who participate in crowdsourcing activities report altruistic motivations, hoping to help their community or the public at large (DREAM Challenges, 2019; Mathews et al., 2018; Zhang et al., 2017b). Including a sharing component fulfills this obligation to give back to the public.

First, an organization has a group (including those with skills and those without skills) attempt to solve a problem. The group could be working independently or collaborating as a team. The rationale for sourcing solutions from a group rather than select individuals includes the following: (1) the potential for groups to have relevant knowledge and experiences in a related field; (2) the importance of public participation and community consultation in health services; (3) the potential for local end-users, patients, and others to be more actively engaged in the process of developing new ideas; (4) the inclusion of people from the community to assist in designing interventions that would be feasible and relevant in the local community. The group participation component of crowdsourcing has been used by states, international organizations, and non-profits for centuries. For example, in 1714, the British government wanted to find an accurate method to measure a ship’s longitudinal position. They offered a cash prize to whomever developed a solution that met pre-specified benchmarks. This spurred many groups to focus on enhanced methods for measuring longitude, resulting in important advances in this field (McKinsey, 2009).

The second key component of crowdsourcing involves sharing solutions. This could be accomplished through implementing the solution in a local community (Tang et al., 2018) or creating open access materials for public use (Wu et al., 2018). For example, the rights to an exceptional crowdsourced image could be made widely available through creative commons attribution. Crowdsourcing approaches may generate a range of materials and products that can be shared in both digital and in-person formats. Some examples of ways that crowdsourced materials have been shared include: providing crowdsourced images, concepts, and logos to the public through an open access website; (Wu et al., 2018) widely distributing images through social media; (Zhang et al., 2015) evaluating the effectiveness of the crowdsourced output through a trial; (Tang et al., 2016a, 2016c, 2018) holding a series of in-person workshops to communicate crowdsourced findings with key stakeholders (Zhang et al., 2017a).

These two crowdsourcing components—group participation and sharing solutions—are each indebted to earlier multidisciplinary concepts on collective intelligence and open source models, respectively. The next two section explores these related concepts as they inform crowdsourcing.

### Collective intelligence

Collective intelligence suggests that in certain settings, a group is better able to solve difficult problems than an individual working alone. The concept is not a universal statement about groups being wiser than individuals, but rather that there are certain contexts wherein this is true. The collective intelligence concept has a history in political science, philosophy, social science, and biology. Perhaps the earliest mention of this concept was in 1785 when Marquis de Condorcet published a theorem about the relative probability of a given group of individuals arriving at a correct decision (De Condorcet, 1785). The theorem examines the optimal number of voters when engaging in a group decision. The number is greater when there is a higher probability of each voter making a correct decision; the number is small when there is a lower probably of each voter making a correct decision. This provides a theoretical basis for democracy and has been widely used in political science (Austen-Smith & Banks, 1996; Ladha, 1992). Within a health context, Condorcet’s theorem has been used in clinical diagnostic imaging (Gottlieb & Hussain, 2015) and reviewing organ transplant eligibility (Koch & Ridgley, 2000).

Philosophers and others have contributed to the development of a collective intelligence concept. The French philosopher Lévy (1997) defined collective intelligence as “a form of universally distributed intelligence, constantly enhanced, coordinated in real time, and resulting in effective mobilization of skills.” Social reformers have also used collective intelligence as a key guiding principle. Wells (1938) described a “World Brain” concept that would help citizens to share information as a group, benefiting from local knowledge and experience within a common platform. He envisioned the platform as a non-commercial resource that would span political boundaries and help expand knowledge (Wells, 1938). The crowdsourced encyclopedia, Wikipedia, echoes some of the structures and functions of Wells’ original world brain concept.

Empirical evidence from humans suggests that in some contexts, a convergent collective intelligence factor explains a group’s performance on several tasks (Woolley et al., 2010). Further empirical evidence supporting collective intelligence is summarized in Surowiecki’s (2004) *The Wisdom of Crowds*. He argues that four elements are necessary for collective intelligence—diversity of opinion, independence of individual ideas, decentralization of ideas, and a way to aggregate individual ideas. Surowiecki shows how collective intelligence has been used in many different contexts, ranging from prediction markets to the Delphi method. The Delphi method has a group of individuals iteratively answer questions and converge on a single answer. The method has been widely used to achieve group consensus in health guidelines (Diamond et al., 2014).

Collective intelligence approaches have been evaluated in several medical settings. Research among medical students suggests that groups of medical students have increased diagnostic performance compared to individual medical students (Hautz et al., 2015; Kämmer et al., 2017). Similar approaches have been evaluated in the context of physician diagnosis of skin cancer (Kurvers et al., 2016) and breast cancer (Wolf et al., 2015).

### Open source model

Open source models can inform the second important component of crowdsourcing—sharing solutions. Open source refers to a decentralized structure that facilitates collaboration and online sharing. Open source models were developed in the 1960s and 1970s as a way to collaboratively develop software and share code (Von Hippel & Von Krogh, 2003). In 1969, the United States Advanced Research Project Agency created the first large, high-speed computer network. This extended opportunities for sharing code among broader online groups. For example, the Linux operating system is one of the first open source operating systems, shared online and available for free to anyone. Linus Torvalds developed the source code for this operating system by sending it to other internet users who helped improve it on a volunteer basis. The collective development of open source products, such as Linux, demonstrate how large, diverse groups working together can iteratively enhance a product that is openly available, to the benefit of all.

This trend also led to the development of Creative Commons, a non-profit organization that allows individuals to legally change and share creative works. The organization has a series of copyright licenses that clarify the terms of sharing. There are currently approximately 1.4 billion works that have been licensed through Creative Commons.

Open source models have increasingly appeared in medicine. For example, several drug development projects have used open source models (Bombelles & Coaker, 2015; Munos, 2006, 2010; So et al., 2011). A project called open source pharma focuses on developing drugs through open source methods. Thousands of volunteers from over 100 countries have helped with micro-tasks to develop more effective drugs for tuberculosis, schistosomiasis, and other infectious diseases (Bhardwaj et al., 2011). The open source platform has resulted in high-quality research, including advances related to the development of schistosomiasis drugs (Årdal & Røttingen, 2012). Other open source models for drug discovery have been developed for Huntington’s disease (Wilhelm, 2017), malaria (Årdal & Røttingen, 2015), eumycetoma (Lim et al., 2018), and other diseases (Bagla, 2012).

Open source models have also been used within genomics. A Shiga-toxin producing *E. coli* outbreak occurred in Germany in 2011, infecting 3,000 individuals. Scientists used an open source model to organize the analysis of a genome sequence from a single individual. The collaborative effort brought together volunteers from around the world, creating the genome sequence within 2 weeks of receiving the DNA samples (Rohde et al., 2011). In addition, the DREAMS Challenge team has organized many open source innovation challenges (Saez-Rodriguez et al., 2016). These typically involve volunteers collaboratively working together to solve a problem related to big data and genomics. Several evaluations of this approach have found it to be effective in developing prognostic models based on clinical data (Allen et al., 2016; Guinney et al., 2017; Noren et al., 2016). Both collective intelligence and open source models reveal some of the theoretical antecedents of crowdsourcing.

### Relationship to other research approaches

Crowdsourcing as an approach is distinct from, but related to community-based participatory research, participatory action research, and community-driven research. Each of these different approaches has a conceptual framework, methods, and assumptions. At the same time, each of these three approaches can be used to inform medical research.

Community-based participatory research actively engages the community in all stages of the research process, contributing to shared decision making and community ownership (Minkler & Wallerstein, 2003). The community plays a central force in setting the agenda, implementing the study, and evaluating the results, such that local community members and researchers iteratively collaborate to improve the health of the community. Similarities between community-based participatory research and crowdsourcing include the following: a focus on listening to and partnering with local communities; a potential to increase healthy equity; an acknowledgement that communities can be a powerful source of new ideas. These areas of convergence suggest that community-based participatory research could be a useful complement to crowdsourcing. For example, community-based participatory research was used to increase community engagement in an HIV cure research project (Mathews et al., 2018).

Other related approaches include participatory action research and youth participatory action research. Participatory action research focuses on partnering with communities to participate in research and achieve social change (Bradbury, 2015). Youth participatory action research provides youth with opportunities to learn about social problems that affect their lives and then propose actions to address these problems (Cammarota & Fine, 2008; Kirshner, 2010; Ozer et al., 2016). The participatory action approach considers youth as potential experts and co-creators of knowledge (Ozer, 2016). Shared elements of crowdsourcing and participatory research approaches include the emphasis on participation, local community partnerships, and empowerment of the public. Participatory action research has been used to complement crowdsourcing projects related to environmental health (English, Richardson & Garzón-Galvis, 2018) and to design crowdsourcing approaches for HIV self-testing (ITEST, 2018).

Finally, community-driven research is another approach related to crowdsourcing. Community-driven research has community members and researchers collaboratively design, implement, analyze, interpret, and disseminate research findings (Orionzi et al., 2016). Community-driven research starts with an assessment of local priorities from the perspective of the community. Both community-driven research and crowdsourcing focus on community-led research, developing ideas and programs from the bottom-up for the community (McElfish et al., 2015). All three of these approaches have been used in health research. We now turn to examine crowdsourcing specifically in the context of health.

### Critiques of crowdsourcing

There are three main critiques of crowdsourcing that merit consideration—the madness of groups concept, the problem of low-quality submissions, and cognitive fixation on examples. We will examine each of these critiques generally and then in the context of crowdsourcing as it applies to medicine.

First, the madness of groups refers to the potential for groups to create and disseminate popular delusions, contributing to panic and moral outrage (Mackay, 1852). The 19th century journalist Charles Mackay remarked, “Men, it has been said, think in herds; it will be seen that they go mad in herds, while they only recover their senses slowly, and one by one.” Psychologists have examined how individual behaviors contribute to and diverge from the collective behavior of the groups. Group behavior may be associated with a loss of responsibility. This is illustrated in the case of Boaty McBoatface, a boat name chosen from a public online poll in the United Kingdom. This name was the most popular in the #NameOurShip poll, but ultimately not used to name the ship (Ellis-Petersen, 2016). One example of mad crowds in the context of medicine is low vaccine uptake. Several negative social media reports that spread through online networks have influenced vaccine uptake and disease outbreaks (Larson et al., 2013).

However, crowdsourcing as an approach does not suggest that all groups are wise at all times, but rather that there are specific conditions that can allow for wise groups. In addition, several individuals have made rebuttals and clarified the concept of a mad group. McPhail (1991) has shown how mad groups are primarily the result of individuals, rather than a group disposition. Empirical data on whether group behavior results in a loss of responsibility has been mixed (Manstead & Hewstone, 1995). Within the context of medicine, online platforms have propagated myths and misunderstandings about disease (Lavorgna et al., 2017; Powell et al., 2016). Submissions to innovation challenges may include myths (Mathews et al., 2018), but judging typically finds these submissions of lower quality. Other ways to limit the risk of mad crowds is to have multi-phase challenges with vetting (Fitzpatrick et al., 2018) or online moderation of submission platforms (Rice et al., 2016).

Second, crowdsourcing projects are sometimes associated with many low-quality outputs. A systematic review of crowdsourcing suggests that only a subset of outputs are excellent (Pan et al., 2017). Having those without formal training contribute to a more complex medical project will result in a wide range of outputs, especially when mass engagement translates into hundreds of submissions. However, the ability to prompt a large number of submissions is an advantage of crowdsourcing and suggests that a wider group of individuals is actively participating. Several techniques for judging have been developed to assess large numbers of crowdsourcing contributions (Han et al., 2018), including group judging (having a group of individuals evaluate) (Tang et al., 2018), panel judging (having a diverse group of individuals evaluate) (Zhang et al., 2015), and artificial intelligence (Albarqouni et al., 2016; Mudie et al., 2017). Several systematic reviews of crowdsourcing in medicine suggest that crowdsourcing allows a broad range of quality, including both low and high-quality submissions (Crequit et al., 2018; Dai, Lendvay & Sorensen, 2017; Ranard et al., 2014).

Finally, the problem of cognitive fixation on prior ideas has been described in crowdsourcing (Fu et al., 2017). This refers to the phenomenon when providing an example or reference limits the diversity of ideas solicited. This concept is similar to groupthink, which occurs when a group of individuals converges on a single solution (Janis, 1972). There are several technical ways of designing a crowdsourcing project that could limit cognitive fixation, including the following: limiting the use of examples when calling for innovative ideas; drawing on different groups of individuals or different topics (avoiding serial challenges focused on the same topic); and having a submission system in which those who submit do not view other submissions.

### Crowdsourcing applications in medical research

Crowdsourcing approaches have already been used to enhance formative, pre-clinical, and clinical research (Table 3). Crowdsourcing approaches have been used to assist in the discovery and development of antibiotics (Desselle et al., 2017), lupus drugs (Grammer et al., 2016), and anti-malarials (Spangenberg et al., 2013). Several crowdsourcing activities have been used to prepare for clinical and other medical research. Crowdsourcing approaches have identified potentially relevant citations as part of systematic reviews. This approach has been found reliable (Mortensen et al., 2017) and is being piloted as part of a Cochrane program (Cochrane Collaboration, 2019).

**Table 3:** Crowdsourcing applications in medical research.

Crowdsourcing application	Purpose of crowdsourcing	Examples
Informing medical research (formative)	Optimize search processes	Assist with systematic reviews
Pre-clinical research	Share key elements necessary for drug development	Curate data on drugs; accelerate genomic analysis
Clinical and translational research	Recruiting study participants; community engagement	Solicit community feedback; enhance drug development

**Note:**

Crowdsourcing can be used to inform formative work, pre-clinical research, and clinical research.

Crowdsourcing could accelerate several stages of drug development, including screening, pre-clinical trials, and human clinical trials. Screening of potential drug candidates has been opened to the public through crowdsourcing activities in several fields. The Medicines for Malaria Venture (Spangenberg et al., 2013) and a tuberculosis consortium (Ballell et al., 2013) both used crowdsourcing to catalyze drug target identification. At the pre-clinical stage of drug development, sharing of chemical probes with the public has created a new class of bromodomain inhibitors (Arshad et al., 2016; Scott, 2016). Within human trials, several studies have used crowdsourcing to develop human clinical trial study messaging and community engagement (Leiter et al., 2014; Mathews et al., 2017; Pan et al., 2017). Many studies have used Amazon Turk or other platforms to recruit study participants into online randomized controlled trials (Jones et al., 2013; Losina et al., 2017; Tang et al., 2016a, 2016b). While such approaches are often rapid and save money, there are concerns about generalizability (Wang et al., 2018b).

## Conclusion

Our observations about using crowdsourcing in medical research have several important limitations. First, we did not focus our analysis based on different categories of crowdsourcing because other systematic reviews have covered this territory (Crequit et al., 2018; Wang et al., 2018a). Second, although there is a growing literature on crowdsourcing in medical research, (Pan et al., 2017) the number of randomized controlled trials and related studies is still limited (Wang et al., 2018a). Third, we have not included a list of areas which problems may be more amenable to crowdsourcing because this has been partially covered in a previous review (Wazny, 2017) and is difficult to infer from the existing literature.

This review suggests several important areas for future crowdsourcing research in medicine. More rigorous research studies are needed to expand our understanding of crowdsourcing, including studies with comparator groups (e.g., randomized controlled trials), cost-effectiveness research, and qualitative studies. In addition, given that much of the crowdsourcing medical research to date has benefitted from academic medical schools as innovation hubs (Siefert et al., 2018), further development of crowdsourcing in medical training and education may be warranted. The design of innovation challenges is widely known among practitioners to influence the ultimate success of crowdsourcing activities, but these design elements are not frequently captured in studies. Further methodological innovation and research are needed.

## Additional Information and Declarations

### Data Availability

The following information was supplied regarding data availability: This article did not generate raw data; this is a literature review.
